# Correction: Mouse model of SARS-CoV-2 reveals inflammatory role of type I interferon signaling

**DOI:** 10.1084/jem.2020124102192025c

**Published:** 2025-02-26

**Authors:** Benjamin Israelow, Eric Song, Tianyang Mao, Peiwen Lu, Amit Meir, Feimei Liu, Mia Madel Alfajaro, Jin Wei, Huiping Dong, Robert J. Homer, Aaron Ring, Craig B. Wilen, Akiko Iwasaki

Vol. 217, No. 12 | https://doi.org/10.1084/jem.20201241| August 4, 2020

The authors regret that the original article contained typographical errors in the Materials and methods regarding the dosing and administration of anesthetics and analgesics. The revised “AAV infection” section is provided here, with corrected text in bold. The errors appear in print and in PDFs downloaded before February 21, 2025.

## AAV infection

AAV9 encoding hACE2 was purchased from Vector Biolabs (AAV-CMV-hACE2). Animals were anaesthetized using a mixture of ketamine (**100 mg/kg**) and xylazine (**10 mg/kg**), injected intraperitoneally. The rostral neck was shaved and disinfected. A 5-mm incision was made, the salivary glands were retracted, and the trachea was visualized. Using a 32-G insulin syringe, a 50-µl bolus injection of 10^11^ genomic copies per milliliter of AAV-CMV-hACE2 or control (AAV-GFP or PBS) was injected into the trachea. The incision was closed with 4–0 Vicryl suture. Following **subcutaneous** administration of analgesic (**5 mg/kg** meloxicam and **3.25** mg/kg buprenorphine **XR**), animals were placed in a heated cage until full recovery.

